# Globus Pallidus Interna in Tourette Syndrome: Decreased Local Activity and Disrupted Functional Connectivity

**DOI:** 10.3389/fnana.2016.00093

**Published:** 2016-10-14

**Authors:** Gong-Jun Ji, Wei Liao, Yang Yu, Huan-Huan Miao, Yi-Xuan Feng, Kai Wang, Jian-Hua Feng, Yu-Feng Zang

**Affiliations:** ^1^Laboratory of Cognitive Neuropsychology, Department of Medical Psychology, Anhui Medical UniversityHefei, China; ^2^Department of Psychology, School of Education, Hangzhou Normal UniversityHangzhou, China; ^3^Center for Cognition and Brain Disorders and the Affiliated Hospital, Hangzhou Normal UniversityHangzhou, China; ^4^Zhejiang Key Laboratory for Research in Assessment of Cognitive ImpairmentsHangzhou, China; ^5^Collaborative Innovation Centre of Neuropsychiatric Disorders and Mental HealthHefei, China; ^6^Center for Information in BioMedicine, Key Laboratory for Neuroinformation of Ministry of Education, School of Life Science and Technology, University of Electronic Science and Technology of ChinaChengdu, China; ^7^Department of Psychiatry, The Second Affiliated Hospital of Medical College, Zhejiang UniversityHangzhou, China; ^8^Department of Pediatrics, the Second Affiliated Hospital, School of Medicine, Zhejiang UniversityHangzhou, China; ^9^Department of Neurology, The First Affiliated Hospital of Anhui Medical UniversityHefei, China

**Keywords:** resting-state, functional MRI, Tourette’s syndrome, deep brain stimulation, amplitude of low frequency fluctuation, globus pallidus

## Abstract

Globus pallidus interna (GPi) is an effective deep brain stimulation site for the treatment of Tourette syndrome (TS), and plays a crucial role in the pathophysiology of TS. To investigate the functional network feature of GPi in TS patients, we retrospectively studied 24 boys with ‘pure’ TS and 32 age-/education-matched healthy boys by resting state functional magnetic resonance images. Amplitude of low-frequency fluctuation (ALFF) and functional connectivity were used to estimate the local activity in GPi and its functional coordinate with the whole brain regions, respectively. We found decreased ALFF in patients’ bilateral GPi, which was also negatively correlated with clinical symptoms. Functional connectivity analysis indicated abnormal regions within motor and motor-control networks in patients (inferior part of sensorimotor area, cerebellum, prefrontal cortex, cingulate gyrus, caudate nucleus, and brain stem). Transcranial magnetic stimulation sites defined by previous studies (“hand knob” area, premotor area, and supplementary motor area) did not show significantly different functional connectivity with GPi between groups. In summary, this study characterized the disrupted functional network of GPi and provided potential regions-of-interest for further basic and clinical studies on TS.

## Introduction

Tourette syndrome (TS) is a childhood-onset movement disorder characterized by multiple motor and vocal tics lasting longer than 1 year (Association, 2000) and frequently complicated by neurobehavioral comorbidities such as attention deficit hyperactivity disorder (ADHD) and obsessive-compulsive disorder (OCD). In most TS patients, tic severity usually increases during pre-puberty but remits in late adolescence or early adulthood (Leckman, 2003). However, a substantial number of patients exhibit poor response to behavioral and pharmaceutical therapies or experience intolerable side-effects, and thus continue to experience disabling symptoms in adulthood (Muller-Vahl, 2013).

For the intractable TS patients, surgical approaches such as deep brain stimulation (DBS) are effective treatment options. DBS is a reversible therapeutic technique that delivers continuous stimulation to specific neuroanatomical structures through intracranial electrodes connected to a subcutaneous pulse generator. Bilateral centromedian-parafascicular complex (CM/Pf) and globus pallidus interna (GPi) are two of the most frequently selected stimulation targets for DBS treatment of intractable TS. Supportive evidence for the efficacy of these targets, however, is based mainly on non-blinded (Servello et al., 2008) or small sample studies (Ackermans et al., 2011). Recently, a randomized, double-blind, crossover trial indicated that DBS of GPi could significantly reduce tic severity (Kefalopoulou et al., 2015), suggesting GPi as an effective stimulation target for the treatment of TS. GPi is the main output for the basal ganglia. It could be roughly divided into anteromedial and posterolateral parts. The anteromedial part is connected with limbic cortical regions (orbitofrontal cortex, medial prefrontal cortex, hippocampus, amygadala, and anterior cingulate cortex), while the posterolateral part is connected with motor areas (primary motor cortex, premotor cortex, supplementary motor area [SMA]) (Draganski et al., 2008; Manes et al., 2014). Current DBS studies on TS patients suggested that the anteromedial GPi is a preferable target over the posterolateral part (Welter et al., 2008; Sachdev et al., 2014; Schrock et al., 2015; Smeets et al., 2016), although more cases are needed for validation.

Given the clinical efficacy of DBS on GPi, we hypothesized that GPi is a crucial node involving in the pathological changes in TS brain. In this study, we aimed to investigate the local activity and functional network of GPi in TS patients as compared to controls. We also paid particular attention in other three regions-of-interest (ROIs): motor, premotor, and SMA. All these ROIs have been investigated in TS patients by transcranial magnetic stimulation (TMS) (Edwards et al., 2008), but only SMA stimulation showed possible efficacy in improving symptoms (Le et al., 2013; Wu et al., 2014). Notably, we further divided SMA into SMA proper and preSMA, which showed distinct connectivity features (Lehericy et al., 2004). According to a recently proposed “Connected Invasive and Noninvasive Effective Targets (CINET)” theory, we predicted significant functional connectivity between GPi and SMA, but not other TMS targets (Fox et al., 2014). To minimize the influence of potential confounding factors, we included only boys and further excluded patients with comorbid ADHD or OCD to investigate a cohort of “pure” TS patients.

## Materials and Methods

### Subjects

We initially recruited 80 children with TS from the Second Affiliated Hospital of Zhejiang University School of Medicine, China. Clinically assessment for each patient was performed by an experienced neurologist using diagnostic procedures suggested by the Chinese Pediatric Society, Chinese Medical Association. The Yale Global Tic Severity Scale (YGTSS) (Leckman et al., 1989) was used to assess current tic severity. Swanson Nolan and Pelham Scale, and Conner Parents Scale were used to assess ADHD symptoms, and the Yale–Brown Obsessive Compulsive Scale was used to quantify obsessive-compulsive symptoms. All these patients met DSM-IV-TR (Diagnostic and Statistical Manual of Mental Disorders, text revision, 4th edition) criteria for TS. For the purpose of this retrospective study, additional inclusive and exclusive criteria were required. The inclusion criteria were (i) male, (ii) less than 18 years old, and (iii) available multimodal imaging datasets (structure, function, and diffusion images). Exclusion criteria were (i) any other neurological or psychological diagnosis except TS, (ii) structural abnormalities on visual inspection of structural imaging, or (iii) ADHD or/and OCD. Finally, 24 boys with ‘pure’ TS fulfilled all the criteria. Thirty-two age-/education-matched healthy boys were also recruited as the control group. These subjects had no history of neurologic or psychiatric illnesses and no gross abnormalities on brain magnetic resonance (MR) images. All patients and controls were right handed as suggested by the Edinburgh handedness inventory.

After a complete description of the study, informed consent was obtained from all parents according to the Declaration of Helsinki. The study protocol was reviewed and approved by the Local Medical Ethics Committee of the Center for Cognition and Brain Disorders, Hangzhou Normal University, China.

### Imaging Protocol

All imaging data were acquired on a 3.0-Tesla MRI scanner (GE Discovery 750 MRI, General Electric, Milwaukee, WI, USA) at the Center for Cognition and Brain Disorders, Hangzhou Normal University. Foam padding was used to minimize head motion for all subjects. Functional images were acquired using a gradient-recalled echo planar imaging sequence (TR = 2000 ms, TE = 30 ms, and flip angle = 90°). Thirty transverse slices (field of view = 220 mm × 220mm, matrix = 64 × 64, slice thickness = 3.2 mm, no inter-slice gap, and 240 volumes) aligned along the anterior commissure-posterior commissure line were acquired. Subjects were instructed simply to rest with their eyes closed, not to think of anything in particular, and not to fall asleep. Subsequently, 3D T1-weighted anatomical images were acquired in the sagittal orientation using a magnetization prepared rapid acquisition gradient-echo sequence (TR = 8.06 ms, TE = 3.136 ms, flip angle = 8°, field of view = 256 mm × 256mm, matrix = 256 × 256, slice thickness = 1 mm, no inter-slice gap, and 176 slices). Finally, susceptibility-weighted imaging (SWI) was acquired by enhanced T2 star weighted angiograph (TR = 78 ms, TE = 45 ms, flip angle = 15°, slice thickness = 2 mm, inter-slice gap = 1 mm, FOV = 24 mm × 24mm, matrix = 384 × 320). SWI images were used to produce a population based GPi atlas specific to this study. However, due to serious head motion, the quality of SWI was only acceptable in 14 patients and 15 controls. After each scanning session, the responsiveness of the subjects was tested by vocal communication to determine whether they had fallen asleep during the scan.

### Regions-of-Interest Definition

**FIGURE 1:**
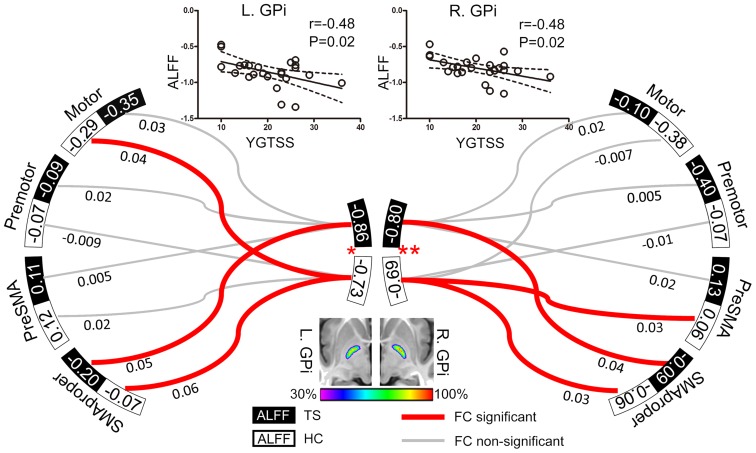
**Resting GPi hypofunction and preserved GPi-SMA proper connectivity in TS patients.** Middle bottom image illustrates the population-based probabilistic map for bilateral GPi in 29 subjects (14 patients and 15 controls). Local resting activity was lower in bilateral GPi of TS patients than matched controls (^∗^*P* < 0.05, ^∗∗^*P* < 0.01). Significant functional connectivity was found between SMA proper and GPi in bilateral hemispheres in both patient and control groups, with no significant between-group difference. The ALFF values of bilateral GPi were significantly correlated with YGTSS, an index of TS severity. The dash line represents the 95% confidence band of the best-fit line. Values nearby curves represent correlation coefficient after Fisher’s *z* transformation.

According to previous TMS studies on TS, four targets (primary motor area, premotor area, SMA proper, and preSMA) were included as regions-of-interest (ROIs) to assess their functional relationships with the GPi (**Figure 1**). ROIs in primary motor area were defined as spheres of 10-mm radius centered on the bilateral “hand knob” area (Montreal Neurological Institute [MNI] coordinates: *x* = 33, *y* = -31, *z* = 57 and *x* = -33, *y* = -31, *z* = 57) (Munchau et al., 2002; Chae et al., 2004; Tinaz et al., 2014). ROIs in bilateral premotor cortex were defined 3 cm anterior to the “hand knob” area (MNI coordinates: *x* = 33, *y* = -1, *z* = 57 and *x* = -33, *y* = -1, *z* = 57; radius = 10 mm) (Munchau et al., 2002; Orth et al., 2005). The bilateral SMA was defined by the Anatomical Automatic Labeling template (Tzourio-Mazoyer et al., 2002) and divided into SMA proper and pre-SMA by a vertical line from the anterior commissure (Johansen-Berg et al., 2004). Two investigators (G-J.J. and W.L.) independently traced the bilateral GPi manually on the susceptibility weighted images (SWIs) of 29 subjects, creating individual volumes of interest (VOIs). The degree of VOI overlap between investigators was high as estimated by the Dice coefficient (mean ± SD: 0.91 ± 0.06). All these VOIs were transformed into MNI space as functional images to create a population-based probability map (**Figure 1**). In this map, voxels shared by more than 30% of subjects were included as the ROI for the GPi.

### Data Processing

#### fMRI Data Preprocessing

Functional MR images (fMRI) were preprocessed using the Data Processing Assistant for Resting-State Functional MR Imaging toolkit (Chao-Gan and Yu-Feng, 2010), which synthesizes procedures in the Resting State Functional MR imaging toolkit (REST^1^) (Song et al., 2011) and SPM8^2^. The first 10 images were excluded to ensure steady-state longitudinal magnetization, and the remaining images were then corrected for temporal differences and head motion. For all the subjects, neither translation nor rotation parameters in any given data set exceeded ±3 mm or ±3°. We then coregistered the individual T1 images to functional images. The T1 images were segmented (gray matter, white matter, and cerebrospinal fluid) and normalized to MNI space using a 12-parameter non-linear transformation. These transformation parameters were also applied to the functional images. The normalized functional images were resampled to 2 mm × 2 mm × 2 mm.

#### Local Activity

The amplitude of low-frequency fluctuations (ALFF) was defined as the averaged square root of activity in the low-frequency band (0.01–0.08 Hz) (Zang et al., 2007). The ALFF value of each voxel was calculated based on the preprocessed data and standardized by a Z transformation (minus mean and divided by standard deviation). Before statistical analysis, ALFF maps were smoothed with a 4-mm full-width at half-maximum isotropic Gaussian kernel. The ALFF values in ten ROIs (bilateral GPi, motor area, premotor area, SMA proper, and pre-SMA) were compared between groups by two-sample *t*-tests.

#### Functional Connectivity

In addition to these preprocessing steps, several possible sources of spurious variance were removed from each voxel by (i) correction for linear trends, (ii) elimination of low-frequency noise using temporal band-pass filtering (0.01–0.08 Hz), and iii) regressing out nuisance variables, including the 24 head-motion parameters, global signal, ventricular signal, and white matter signal. We did not remove the image frames based on head motion (Power et al., 2012) because a recent examination of the relationship between head motion and connectivity indicated that data scrubbing may cause inflated connectivity estimates (Zeng et al., 2014).

We first analyzed the functional connectivity between GPi and TMS targets (motor, premotor, SMA proper, and preSMA) by Pearson’s correlation in each hemisphere. Beside this hypothesis-based analysis, we also computed the functional connectivity of bilateral GPi in the whole brain by Pearson’s correlation. After Fisher’s Z transformation and spatial smooth (with a 4-mm full-width at half-maximum isotropic Gaussian kernel), one-sample *t*-test was used to identify regions where activity was significantly correlated with that in the GPi for each group. The result maps were corrected by an AlphaSim program (cluster size > 410 voxels, signal voxel *P* < 0.05). Only survived voxels were included for the following comparison between groups by two-sample *t*-tests (cluster size > 237 voxels, signal voxel *P* < 0.05; AlphaSim corrected). We performed separate functional connectivity analysis in the whole brain for left and right GPi. Pearson correlation analysis was performed between YGTSS and functional connectivity values at the peak voxel of clusters from the between-group analysis.

Given the clinical efficiency of DBS on the anteromedial part of GPi, we also compared the whole brain functional connectivity of this sub-region between patients and controls. But the findings should be taken in caution since the spatial resolution (3.4 mm × 3.4 mm × 3.2mm) of our datasets may be not high enough for characterizing the functional feature of this small sub-region. See the detailed methods in Supplementary Materials.

## Results

### Clinical Characteristics

From the large dataset (*n* = 80) of our ongoing research project, 24 boys with ‘pure’ TS (without ADHD or/and OCD co-morbidity) and 32 age-, sex-, and education-matched controls were included in the current study. All subjects were right-handed and there was no significant difference in age (*t* = 0.50, *P* = 0.62) or years of education (*t* = 0.18, *P* = 0.86) between groups. The details of each TS patient are given in **Table 1**.

**Table 1:** Demographic and clinical details of the study patients.

Patient	Age	Age at tic onset	YGTSS	Years of education	Medication
1	8	7	16	2	NA
2	12	12	15	6	NA
3	8	7	23	3	Oryzanol
4	9	7	10	4	Tiapride
5	7	6	10	1	Tiapride, TCM
6	9	6	23	4	Oryzanol
7	9	5	26	4	Topamax, Haloperidol, Benzhexol
8	9	8	19	4	Tiapride, TCM
9	11	7	25	6	Haloperidol, Benzhexol, TCM
10	9	7	29	3	Oryzanol, TCM
11	9	5	17	3	Haloperidol, Nitrazepam, TCM
12	7	5	14	1	Tiapride, TCM
13	8	4	16	2	TCM
14	15	9	36	8	TCM
15	7	6	18	1	Oryzanol, TCM
16	11	11	24	4	Oryzanol, TCM
17	7	5	13	1	Tiapride
18	9	5	23	3	NA
19	12	11	22	6	Oryzanol, TCM
20	7	5	10	1	Tiapride, TCM
21	13	11	26	8	Tiapride, Nitrazepam, TCM
22	12	7	20	6	NA
23	8	7	26	2	TCM
24	11	9	26	3	NA

*NA, not available; TCM, traditional Chinese medicine; YGTSS, Yale Global Tic Severity Scale.*

### Local Activity

Patients exhibited significantly lower ALFF in both left (*t* = -2.37, *P* = 0.02) and right (*t* = -2.77, *P* = 0.008) GPi than controls, but similar ALFF values in all cortical ROIs (potential TMS sites) compared to controls (All *P* > 0.05, **Figure 1**). In patients, ALFF values in bilateral GPi were significantly and negatively correlated with YGTSS (*r* = -0.48, *P* = 0.02), suggesting that lower local activity in GPi was associated with more severe symptoms (**Figure 1**).

### Functional Connectivity

ROIs-based analysis indicated non-significant correlations between bilateral GPi and the ipsilateral preSMA, motor, and premotor areas in patients (**Figure 1**), while activity in bilateral SMA proper was positively correlated with that in ipsilateral GPi of both patients (*t* = 2.52, *P* = 0.02 for left; *t* = 2.15, *P* = 0.04 for right) and controls (*t* = 4.28, *P* = 0.0002 for left; *t* = 2.10, *P* = 0.04 for right) (**Figure 1**). There were no significant between-group differences in connectivity strengths between GPi and these 4 cortical ROIs. Therefore, we did not examine correlation between connectivity strength and clinical symptom.

Voxel-wise comparisons indicated that patients showing abnormal functional connectivity in motor-related, and limbic areas in bilateral hemispheres, which is consistent with our previous study using the same datasets (Liao et al., 2016).

**FIGURE 2:**
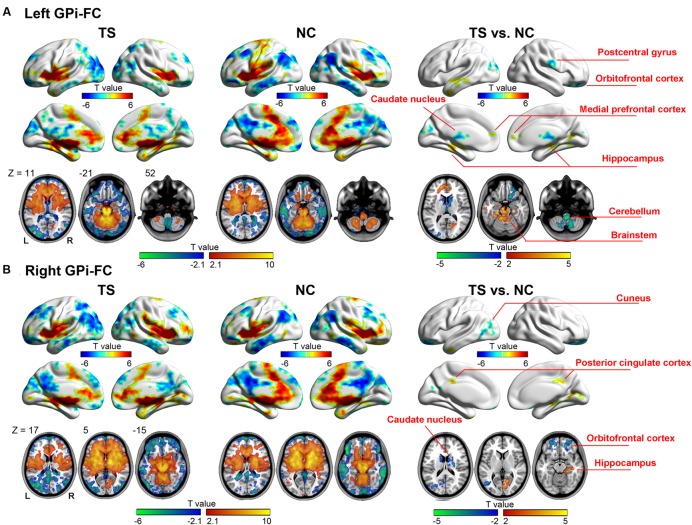
**Whole-brain functional connectivity map of the left (A) and right (B) GPi.** Warm (cold) colors represent positive (negative) correlation with GPi (the left two columns), and increased (decreased) functional connectivity in patients (the right column).

The left GPi showed decreased connectivity with motor-related areas including right pre-/postcentral gyrus, bilateral caudate nucleus, right thalamus, bilateral cerebellum, and left brain stem. The abnormal limbic areas included right obitofrontal cortex (OFC), bilateral medial prefrontal cortex, bilateral anterior cingulate cortex, left parahippucampa gyrus/hippocampus (**Figure 2A**, **Table 2**).

**Table 2:** Regions showing significantly different connectivity with left GPi in TS patients as compared to controls.

MNI coordinate	Brain region	BA	*T* value	Voxel number
68 -8 26	Pre-/postcentral gyrus, R.	4/3	-3.44	328
22 8 20	Caudate nucleus, R., thalamus, R.	NaN	-3.89	967
-16 -22 26	Caudatenucleus, L.	NaN	-3.32	344
-2 -46 -52	Cerebellum, B.	NaN	-4.63	1456
20 44 -16	Obitofrontal cortex, R.	47	-3.63	379
2 50 10	Medial superior frontal gyrus, B., anterior cingulate cortex, B.	10/32	3.04	368
-8 -12 -6	Middle temporal gyrus, L., parahippucampa gyrus/hippocampus, L., brain stem	21/36	4.14	2724
-56 4 -34	Middle temporal gyrus, L.	21	4.27	268
-42 -86 22	Cuneus, L.	18/19	-3.91	968
26 -62 6	Cuneus, R.	19	3.13	284

*B., bilateral hemisphere; BA, Brodmann area; L., left hemisphere; MNI, Montreal neurological institute; R., right hemisphere.*

The right GPi showed abnormal connectivity mainly in limbic areas including the bilateral OFC, right posterior cingulated cortex, right parahippocampa gyrus/hippocampus, and bilateral caudate nucleus. Other abnormal regions included the left middle temporal/occipital gyrus, left middle/inferior temporal gyrus, and right cuneus (**Figure 2B**, **Table 3**). No significant correlation was found between YGTSS score and the abnormal functional connectivity strength (all *P* > 0.05).

**Table 3:** Regions showing significantly different connectivity with right GPi in TS patients as compared to controls.

MNI coordinate	Brain region	BA	*T* value	Voxel number
18 44 -16	Obitofrontal cortex, R.	11/47	-3.69	539
-12 56 -24	Obitofrontal cortex, L.	11/47	-4.32	674
-8 10 12	Caduate, L.	NaN	-3.78	340
10 2 20	Caduate, R.	NaN	-4.12	345
18 -46 32	Posterior cingulated cortex, R.	31	3.56	283
38 -24 -12	Parahippocampa gyrus/hippcampus, R.	28	3.65	337
-34 8 -42	Middle/inferior temporal gyrus, L.	20/21	4.67	337
-50 -66 0	Middle temporal/occipital gyrus, L.	19, 37	-3.21	688
14 -62 4	Cuneus, R.	18	3.64	311

*B., bilateral hemisphere; BA, Brodmann area; L., left hemisphere; MNI, Montreal neurological institute; R., right hemisphere.*

Since the anteromedial GPi has been suggested as the most effective DBS target for TS treatment, we further investigated its functional connectivity feature in TS patients. As expected, both left and right anteromedial GPi showed abnormal connectivity with limbic areas, including the bilateral OFC, hippocampus and subcortical structures (See Supplementary Table E1, and Supplementary Figure E1).

## Discussion

This study investigated the local and functional connectivity feature of GPi in TS patients by resting state fMRI. Decreased local activity was found in both left and right GPi, and negatively correlated with YGTSS score in patients. ROI-based analysis did not find significant between-group difference in functional connectivity between bilateral GPi and TMS targets. Voxel-wise analysis indicated abnormal motor-related and limbic areas. These findings provided fundamental information about the functional feature of the effective DBS target (i.e., GPi) in ‘pure’ TS boys, thus improved our understanding of the role of GPi in the pathophysiology of TS.

### Local Activity

We selected the GPi as the representative “effective site” because the efficacy of GPi-targeted DBS has been demonstrated in a randomized, double-blind, crossover trial (Kefalopoulou et al., 2015). Consistent with *in vivo* electrophysiological studies showing low firing rates below normal non-human primates (Raz et al., 2000; Zhuang et al., 2009), we observed decreased functional activity of bilateral GPi in TS patients. Correlation analysis indicated that symptom severity increased with GPi hypofunction. Thus, our findings provide a systems-level description of GPi dysfunction that bridges the gap between cellular-level features of TS and clinical observations. On the other hand, all cerebral ROIs chosen as potential TMS targets showed normal functional activity in TS patients. Using the same measure (ALFF), a voxel-wise analysis in the whole brain also revealed normal local activity in these ROIs (Cui et al., 2014). Furthermore, a magnetic resonance spectroscopy study also indicated a normal GABA+/Cre ratio in bilateral motor area in adult TS patients (Tinaz et al., 2014).

### Functional Connectivity

According to the “CINET theory” proposed by Fox and colleges, effective TMS and DBS targets may belong to the same brain network defined by resting-state functional MRI (Fox et al., 2014). Such connectivity pattern has been demonstrated in some neurological and psychiatric conditions, such as Parkinson’s disease (PD) and depression (Fox et al., 2012, 2014). However, the integrity of DBS-TMS target connectivity had not been determined in TS patients. Our findings indicate that the effective DBS target (i.e., GPi) has strong functional connectivity with a putatively effective TMS target (i.e., SMA) but not with nearby ineffective TMS sites (i.e., motor and premotor areas) in TS patients. These findings strongly support the CINET theory (Fox et al., 2014) in the context of TS. Additionally, this study shows that the functional integrity between effective DBS and TMS targets in TS patients remains normal as controls.

To explore the whole brain connectivity feature, we further performed a voxel-wise functional connectivity analysis of GPi. The findings are consistent with the ROI analysis and our previous homotopic connectivity study (Liao et al., 2016). We found some motor-related areas showing abnormal connectivity with GPi in patients, such as pre-/postcentral gyrus, prefrontal cortex, cerebellum, and subcortical structures. These regions constitute motor pathways and fronto-striato-thalamo-fronto circuits that exert top-down control over motor pathways (Mink, 2001; Wang et al., 2011). Using different self-regulatory control tasks, the abnormal activation of frontostriatal system has been consistently reported (Marsh et al., 2007; Raz et al., 2009; Mazzone et al., 2010). Other studies regarded tics as events and found widespread brain areas activated even before tics onset (Bohlhalter et al., 2006; Neuner et al., 2014), which partly overlapped with the abnormal regions found in the current study. Some of these abnormal regions may involve the initiation of tic behavior (such as pre-/postcentral gyrus, prefrontal cortex, cingulate gyrus, brain stem and cerebellum), while others may also represent features of the premonitory urges (such as somatosensory, occipital areas and hippocampus complex) (Wang et al., 2011). Grange causality analysis indicated increased interaction within motor pathway (including pre-/postcentral gyrus, basal ganglia, and brain stem) and decreased motor control circuits (including caudate nucleus and cingulate gyrus) (Wang et al., 2011). In addition, we also found abnormal connectivity in limbic areas, such as anterior/posterior cingulate cortex, medial temporal lobe, and OFC. These findings were partly confirmed by our supplementary analysis using anteromedial GPi as seed region. GPi is a key output node of basal ganglia. This GPi-limbic loop involves in reward learning and motivational behavior associating with the TS symptoms (Jahanshahi et al., 2015). As DBS studies have suggested the anteromedial part as the most effective target for TS treatment (Welter et al., 2008; Sachdev et al., 2014; Schrock et al., 2015; Smeets et al., 2016), future longitudinal fMRI researches are needed for understanding the role of GPi-limbic connection in surgical success. Notably, most of the task fMRI evidences are from adults patients. Considering childhood patients have distinct pathophysiology to adults (Raz et al., 2009), some studies paid particular attention on childhood patients, and also found abnormal function in motor control network (Church et al., 2009; Cui et al., 2014; Ganos et al., 2014). Complementary to these studies, our findings characterized the baseline brain function in ‘pure’ TS boys, suggesting the effective DBS site (GPi) as a crucial region in the abnormal motor control network.

Transcranial magnetic stimulation is a non-invasive brain stimulation technique, and has been applied in a variety of neurological disorders, such as TS and PD. But the clinical effectiveness varied with subjects (Le et al., 2013; Wu et al., 2014). Among a number of factors, the importance of target selection has been emphasized (Fox et al., 2012). As predicted by the “CINET theory”(Fox et al., 2014), we found significant functional connectivity between GPi and SMA. Additionally, we revealed a number of motor and limbic areas abnormally connected with GPi. Among these regions, we speculated one of the superficial cortexes, the postcentral gyrus, may be an optional target for TMS treatment. As TMS could modulate remote functional connectivity of target (Wang et al., 2014; Valchev et al., 2015), our findings suggest the postcentral gyrus as a key node for relaying the TMS effect to the abnormal whole brain networks of TS patients. Notably, PD has similar DBS target in thalamus as TS. A randomized, double-blind, sham-controlled, multicenter study also provided Class I evidence that the TMS over the SMA is effective for improving motor symptoms in PD. Here, we also found that a number of regions have been reported abnormal in PD, mainly in motor systems (Wu et al., 2011a,b, 2016). Thus, we speculated that they may share a common pathology changes underlying the motor symptoms. Future fMRI studies directly compared these two disorders may help to make this issue more clear.

### Limitation and Future Directions

First, this study is based on a single small cohort of pediatric male patients, so applicability to other TS populations, such as adult patients, is unclear. Second, we adopted functional connectivity to model the relation between GPi and other brain areas. Based on our findings, future studies with modern dynamic and causal model may helpful to illustrate the interaction more clearly. Third, future studies following the development of these childhood patients will provide valuable information about the progression of TS, and connect the evidences between children and adults. Fourth, it is hard to recruit enough drug-naïve patients in practice and most patients in the current study were treated by medication, thus we cannot exclude the medication contribution on our findings. As our study project goes on, we may recruite enough “pure” drug-naïve TS patients after several years.

## Conclusion

In this study, we hypothesized the effective DBS site (GPi) as a crucial region in the abnormal functional network of TS and investigated its functional network in ‘pure’ TS boys by resting state fMRI. We found hypofunction inbilateral GPi, which negatively correlated with symptom severity. Bilateral GPi also indicated abnormal connectivity with motor and limbic networks. Among the abnormal regions, some superficial areas (e.g., postcentral gyrus) may be tested as potential targets for non-invasive treatment (e.g., TMS) on TS according to the ‘CINET’ theory (Fox et al., 2014). In summary, this study characterized the disrupted functional network of GPi and provided potential ROIs for further basic and clinical studies on TS.

## Author Contributions

G-JJ and WL: study design, data analyses and interpretation, manuscript drafting, critical revision of the manuscript for important intellectual content. YY, H-HM, Y-XF, and J-HF: data collection and preliminary analyses of clinical and imaging data. Y-XF, and J-HF: clinical diagnosis for Tourette syndrome patients. KW, J-HF, Y-FZ: study design and supervision, critical revision of the manuscript for important intellectual content.

## Conflict of Interest Statement

The authors declare that the research was conducted in the absence of any commercial or financial relationships that could be construed as a potential conflict of interest.

## Footnotes

1 http://www.restfmri.net

2 http://www.fil.ion.ucl.ac.uk/spm
