# Supplementary material for: Globus Pallidus Interna in Tourette Syndrome: Decreased Local Activity and Disrupted Functional Connectivity
Source: Front Neuroanat. 2016 Oct 14;10:93. doi: 10.3389/fnana.2016.00093 (PMC5064665; doi:10.3389/fnana.2016.00093)
Supplement: Supplementary file 1 [file Data_Sheet_1.docx]

**Supplementary Materials**

**Methods**

We divided the GPi into anteromedial and postolateral sub-regions according to its longest left-right axis. Specifically, the left to right coordinate ranged from X = -22 to -10 and X = 12 to 24 for left and right GPi, respectively. GPi regions with X coordinate smaller than -16 (or larger than 16) were classified as left (or right) anteromedial GPi.

We computed the functional connectivity of bilateral anteromedial GPi in the whole brain by Pearson’s correlation. After Fisher’s Z transformation and spatial smooth (with a 4-mm full-width at half-maximum isotropic Gaussian kernel), one-sample t test was used to identify regions where activity was significantly correlated with that in the anteromedial GPi for each group. The result maps were corrected by an AlphaSim program (cluster size > 410 voxels, signal voxel P < 0.05). Only survived voxels were included for the following comparison between groups by two-sample t tests (cluster size > 235 voxels, signal voxel P < 0.05; AlphaSim corrected). We performed separate functional connectivity analysis in the whole brain for left and right GPi.

**Results:**

Table E1 Regions showing significantly different connectivity with left and right anteromedial GPi in TS patients as compared to controls

| MNI coordinate | Brain region | | BA | | T value | | Voxel  number |
| --- | --- | --- | --- | --- | --- | --- | --- |
| Right anteromedial GPi | | |  | |  | |  |
| -12 56 -26 | Orbitofrontal cortex L. | | 11 | | -5.06 | | 863 |
| 18 44 -16 | Orbitofrontal cortex R. | | 11 | | -4.05 | | 584 |
| 38 -2 -12 | Hippocampus R. | | 35 | | 4.39 | | 313 |
| 62 6 6 | Superior temporal gyrus R. | | 22 | | 3.70 | | 400 |
| -62 -4 8 | Superior temporal gyrus L. | | 22 | | 4.17 | | 340 |
|  |  | |  | |  | |  |
| Left anteromedial GPi | |  | |  | |  | |
| -2 -44 -54 | Cerebellum B. | | NaN | | -4.59 | | 1984 |
| -2 -92 -28 | Cerebellum B. | | NaN | | -3.78 | | 515 |
| -26 -30 -14 | Parahippocampa L. | | 36 | | 3.90 | | 376 |
| 24 40 -20 | Orbitofrontal cortex L. | | 11 | | -4.10 | | 277 |
| -8 -12 -6 | Brainstem L. | | NaN | | 4.58 | | 1076 |
| 26 -62 6 | Calcarine R. | | 30/19 | | 3.88 | | 399 |
| 18 -8 22 | Caudate nucleus B. | | NaN | | -3.93 | | 1182 |

B. = bilateral hemisphere; BA = Brodmann area; L. = left hemisphere; MNI = Montreal neurological institute; R. = right hemisphere.


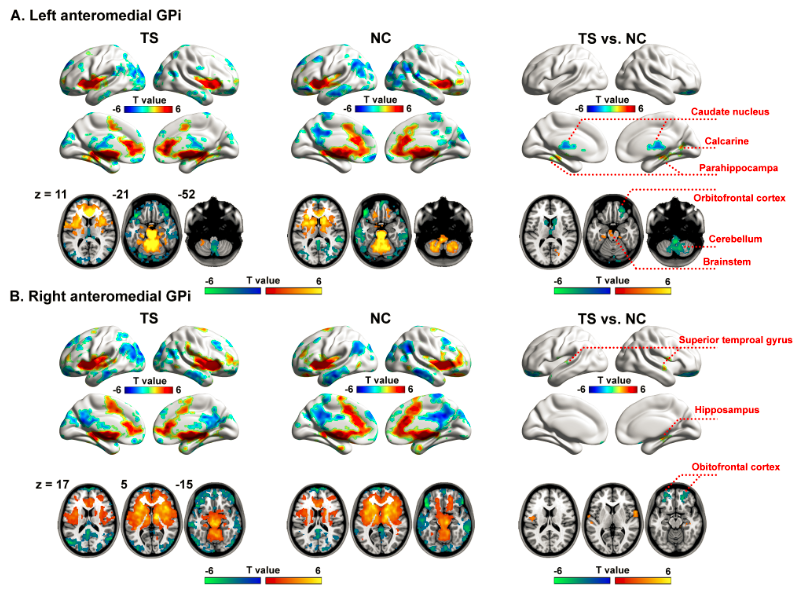


Figure E1. Whole-brain functional connectivity map of the left (A) and right (B) anteromedial GPi. Warm (cold) colors represent positive (negative) correlation with GPi (the left two columns), and increased (decreased) functional connectivity in patients (the right column).
